# Publisher’s Announcement

**Published:** 1881-08

**Authors:** H. H. Dickson

**Affiliations:** 32 Broad St.; Atlanta, Ga.


### PUBLISHERS' ANNOUNCEMENT.

As my contract with Drs. J. G. and R. W. Westmoreland as editors of the JOURNAL, expires with the September number, I desire to announce that after that time the editorial department of the JOURNAL will be under the control of Drs. Jas. B. Baird and Jno. Thad. Johnson. These gentlemen will take charge of the editorial columns of the JOURNAL with the October number, and all matter intended for publication should be addressed to them.

I can safely promise the readers of the JOURNAL that they will in future find its columns more entertaining and instructive than for some time past.

All business communications should be addressed as before to H. H. DICKSON,

Atlanta, Ga., August, 1881. 32 Broad St.

